# Expression profile of SARS‐CoV‐2 cellular entry proteins in normal oral mucosa and oral squamous cell carcinoma

**DOI:** 10.1002/cre2.510

**Published:** 2021-11-02

**Authors:** Dipak Sapkota, Sunita Sharma, Tine M. Søland, Paulo H. Braz‐Silva, Muy‐Teck Teh

**Affiliations:** ^1^ Department of Oral Biology, Faculty of Dentistry University of Oslo Oslo Norway; ^2^ Department of Pathology Rikshospitalet, Oslo University Hospital Oslo Norway; ^3^ Department of Stomatology, School of Dentistry University of São Paulo São Paulo Brazil; ^4^ Laboratory of Virology, Institute of Tropical Medicine of São Paulo, School of Medicine University of São Paulo São Paulo Brazil; ^5^ Centre for Oral Immunobiology and Regenerative Medicine, Institute of Dentistry, Barts and The London School of Medicine and Dentistry Queen Mary University of London London UK; ^6^ China‐British Joint Molecular Head and Neck Cancer Research Laboratory Affiliated Stomatological Hospital of Guizhou Medical University Guiyang China

**Keywords:** ACE2, COVID‐19, expression, head cancer, microarray, neck cancer, oral cancer, tongue

## Abstract

**Objective:**

Besides angiotensin converting enzyme 2 (ACE2), an active involvement of proteases (FURIN and/or TMPRSS2) is important for cellular entry of SARS‐CoV‐2. Therefore, a simultaneous expression profiling of entry proteins in a tissue might provide a better risk assessment of SARS‐CoV‐2 infection as compared to individual proteins. In an attempt to understand the relative susceptibility of oral squamous cell carcinoma (OSCC) lesions as compared to the normal oral mucosa (NOM) for SARS‐CoV‐2 attachment/entry, this study examined the mRNA and protein expression profiles of ACE2, FURIN, and TMPRSS2 in the corresponding tissues using public transcriptomic and proteomics datasets.

**Methods and methods:**

Public transcriptomic and proteomics datasets (the Cancer Genome Atlas (TCGA)/the Genotype‐Tissue Expression (GTEx), the Human Protein Atlas (HPA), and two independent microarray datasets) were used to examine the expression profiles of ACE2, TMPRSS2 and FURIN in NOM and OSCC.

**Results:**

*ACE2*, *TMPRSS2*, and *FURIN* mRNAs were detected in NOM, however, at lower levels as compared to other body tissues. Except for moderate up‐regulation of *FURIN*, expression levels of *ACE2* and *TMPRSS2* mRNA were unchanged/down‐regulated in OSCC as compared to the NOM.

**Conclusions:**

These results indicate that NOM may serve as a possible site for SARS‐CoV‐2 attachment, however, to a lesser extent as compared to organs with higher expression levels of the SARS‐CoV‐2 entry proteins. However, the evidence is lacking to suggest that expression status of entry proteins predisposes OSCC lesions to additional risk for SARS‐CoV‐2 attachment/entry as compared to NOM.

## 1 INTRODUCTION

The 2019 novel coronavirus disease (COVID‐19) was determined to be caused by a RNA virus, the SARS‐CoV‐2 (Zhou et al., 2020). SARS‐CoV‐2 infection, at the cellular level, depends on the relative abundance of surface proteins involved in cellular attachment and entry of SARS‐CoV‐2 into the host cells. The spike (S) protein, one of the structural proteins encoded by the viral genome, is important for the attachment of virus to the host cells. SARS‐CoV‐2 cellular entry has been suggested to be mediated by binding of receptor binding domain (RBD) of the S protein to a cell surface receptor called angiotensin converting enzyme 2 (ACE2) (Zhou et al., 2020). It has been shown that the RBD, although it possesses a high affinity to ACE2, is in fact less accessible to ACE2 binding as compared to the SARS‐CoV RBD, thereby requiring selective cleavage (priming) of the S protein by cell surface proteases (co‐receptors) for successful cellular entry (Shang et al., 2020). FURIN (encoded by *FURIN* gene) is involved in pre‐priming of S protein, thereby facilitating binding of the preactivated S protein with ACE2 (Hoffmann, Kleine‐Weber, & Pöhlmann, 2020; Shang et al., 2020). On the other hand, TMPRSS2 mediates priming of ACE2 bound S‐protein, thereby promoting viral fusion with the host cell membrane (Hoffmann, Kleine‐Weber, Schroeder, et al., 2020). Interestingly, pre‐priming by FURIN and post‐priming by TMPRSS2 proteases have been shown to have a cumulative effect in promoting SARS‐CoV‐2 cellular entry (Shang et al., 2020). The dependency of ACE2 on FURIN and/or TMPRSS2‐mediated priming of the S protein might imply that even a relatively high expression of ACE2 does not always guarantee a successful SARS‐CoV‐2 cellular entry if the expression levels of FURIN and TMPRSS2 are lower or vice‐versa. Hence, a simultaneous expression profiling of ACE2, FURIN, and TMPRSS2 is expected to provide a better risk assessment of SARS‐CoV‐2 attachment on a tissue as compared to the expression status of individual proteins.

Given the key roles of ACE2, FURIN and TMPRSS2 in SARS‐CoV‐2 attachment and cellular entry, tissues with relatively high basal expression of these proteins such as the lung, heart, esophagus, and kidney have been suggested to be high‐risk host tissues for COVID‐19 (Xin Zou et al., 2020). Moreover, it is reasonable to suspect that de‐regulation of these proteins, a frequent observation in human malignancies (Kong et al., 2020), might influence the risk of SARS‐CoV‐2 infection in these lesions as compared to the normal tissues. Based on the *ACE2* mRNA expression in normal/para‐tumor oral epithelium, oral cavity was suggested to be a potential site for SARS‐CoV‐2 attachment (Xu et al., 2020). Similarly, a possible link between oral squamous cell carcinoma (OSCC) and SARS‐CoV‐2 infection was previously hypothesized (Chauhan et al., 2020). However, the expression profiles of ACE2, TMPRSS2 and FURIN have not been comprehensively examined in normal oral mucosa (NOM) and OSCC, a major subgroup of head and neck squamous cell carcinoma (HNSCC). In an attempt to understand the relative susceptibility of NOM and OSCC lesions for SARS‐CoV‐2 attachment, this study examined the mRNA and protein expression profiles of ACE2, FURIN, and TMPRSS2 in the corresponding tissues using public transcriptomic and proteomics datasets.

## 2 MATERIALS AND METHODS

Ethical approval and patient consent was not applicable to the current study, as only the public databases have been used for the analysis.

### 2.1 Transcriptomic datasets

Transcriptomic datasets were obtained from the Cancer Genome Atlas (TCGA)/the Genotype‐Tissue Expression (GTEx) (GTEx, 2015), consensus datasets from the Human Protein Atlas (HPA) (Uhlén et al., 2015), and microarray datasets (Chen et al., 2008; Rentoft et al., 2012). The Consensus mRNA dataset, generated from a combination of three datasets (the HPA RNA‐seq data, GTEx RNA‐seq data [https://www.gtexportal.org/home/], and FANTOM5: the Functional Annotation of Mammalian Genomes 5 data) (Forrest et al., 2014), were obtained from the HPA. The specimen characteristics of different transcriptomic datasets were as follows: the HPA dataset consisted of 483 tissue samples representing 37 different human normal tissues (no oral tissue included), the GTEx consisted of 54 different human tissues across nearly 1000 individuals (no oral tissue included), and the FANTOM5 consisted of specimens from 60 different normal tissues including two tongue specimens. The Consensus mRNA dataset was used to examine the relative mRNA expression levels of *ACE2*, *FURIN*, and *TMPRSS2* in different normal tissues in the body, including the oral cavity. Using the “box plot” tool in GEPIA (Tang et al., 2017), the transcriptomic data from TCGA/GTEx (GTEx, 2015) was used to examine the mRNA levels of *ACE2*, *FURIN*, and *TMPRSS2* in HNSCC (*n* = 519) as compared to the normal/para‐tumor control tissues (*n* = 44). The TCGA/GTEx data in HNSCC were validated using two independent OSCC microarray datasets: Rentoft et al. (2012) consisting of 62 OSCC and 16 NOM, and Chen et al. (2008) consisting of 167 OSCC, 17 oral dysplastic (premalignant oral lesions) lesions and 45 normal oral controls.

### 2.2 Protein datasets

The protein data were obtained from “Protein Expression Overview” tool from the HPA (Uhlén et al., 2015). These data consisted of specimens from 144 individuals corresponding to 44 different normal tissues, with each normal tissue represented by at least three samples. Each bar in the “Protein Expression Overview” tool represents the average intensity of immunohistochemical images for a specific protein across normal tissues.

## 3 RESULTS

### 3.1 mRNA and protein expression levels of ACE2, TMPRSS2, and FURIN in NOM

**Figure 1:**
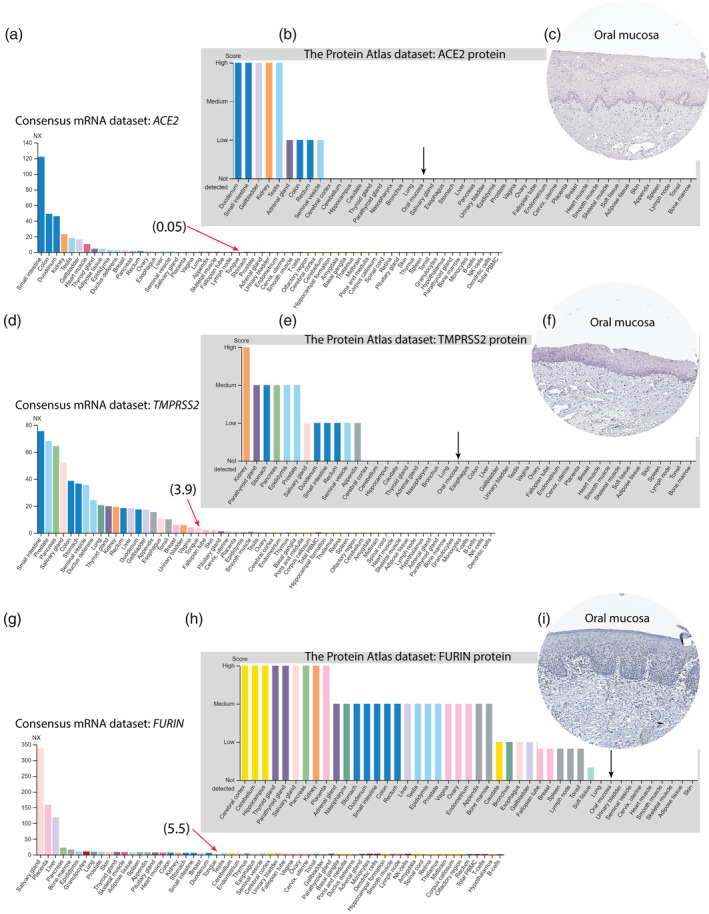
Relative mRNA and protein expression levels of ACE2, TMPRSS2, and FURIN in various normal tissues in the body. The mRNA expression data (a, d, and g) represent the Consensus mRNA dataset obtained from the Human Protein Atlas (HPA). NX, normalized expression. The red arrows indicate mRNA expression levels of the examined genes in tongue. The protein data (b, e, and h) represent the intensity of immunohistochemical images of the proteins examined in different human tissues. The black arrows indicate protein expression levels of the examined protein in oral mucosa. Representative immunohistochemical images of ACE2 (c), TMPRSS2 (f), and FURIN (i) in normal oral epithelium from the HPA dataset

Using the TCGA, the FANTOM5 CAGE, and in‐house data, a previous study reported a relatively high expression of *ACE2* mRNA in normal/para‐tumor oral mucosa (Xu et al., 2020). To further explore the mRNA and protein expression levels of ACE2, TMPRSS2, and FURIN in NOM, expression data from the HPA were analyzed in the current study. The Consensus mRNA dataset from the HPA showed a relatively low (0.05 NX [normalized expression]) *ACE2* mRNA expression in normal tongue as compared to the other organs such as small intestine, colon, and kidney (Figure 1a). However, according to the HPA, no ACE2 protein was immunohistochemically detected in NOM (Figure 1b,c). The mRNA levels of *TMPRSS2* (3.9 NX) and *FURIN* (5.5 NX), although higher than that of *ACE2* (Figure 1d,g) in the normal tongue, were found to be markedly lower than in other normal body tissues. The corresponding protein levels, according to the HPA, were, however, undetectable in NOM (Figure 1e,f,h,i).

### 3.2 mRNA expression levels of *ACE2* , *TMPRSS2*, and *FURIN* in OSCC/HNSCC as compared to the normal controls

**Figure 2:**
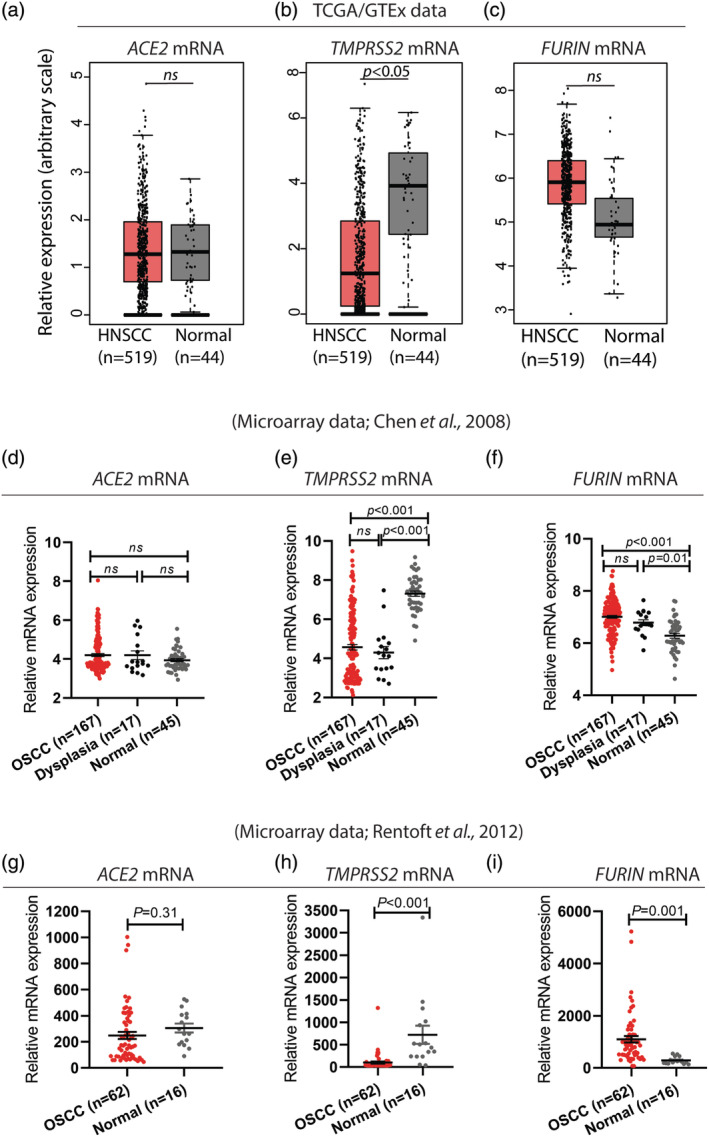
Expression profile of *ACE2*, *FURIN*, and *TMPRSS2* in head and neck squamous cell carcinoma/oral squamous cell carcinoma (HNSCC/OSCC). (a) Expression level of *ACE2* mRNA was similar both in HNSCC and control tissues. *TMPRSS2* mRNA expression (b) was significantly down‐regulated in HNSCC whereas the mRNA expression level of *FURIN* (c) was moderately increased as compared to the control tissues. Publicly available transcriptomic datasets (TCGA/GTEx) were analyzed using the “box plot” tool analysis in GEPIA (Tang et al., 2017). HNSCC (*n* = 519), normal, normal/para‐tumor control epithelium (*n* = 44), ns: non‐significant. (d–i) The HNSCC data were validated using two independent OSCC microarray datasets (d–f: Chen et al., 2008 and g–i: Rentoft et al., 2012). The OSCC cases included in the Rentoft et al., 2012 dataset were all from the tongue, whereas the Chen et al., 2008 dataset consisted of OSCC from different locations in the oral cavity. For (d–f), one‐way analysis of variance analysis with Tukey's multiple comparisons test was used for statistical analysis using GraphPad prism version 8.0.1 for Windows (www.graphpad.com). The error bars represent mean and standard error of the mean. (*n* = 167), Dysplasia: oral dysplastic lesions (*n* = 17), normal, normal control epithelium (*n* = 45). For (g–i), unpaired *t* test was used for statistical analysis using GraphPad prism version 8.0.1 for Windows (www.graphpad.com). The error bars represent mean and standard error of the mean. OSCC (*n* = 62), normal, normal control epithelium (*n* = 16)

Analysis of the TCGA/GTEx data consisting of 519 HNSCC and 44 normal/para‐tumor controls showed that *ACE2* mRNA expression in HNSCC was similar to that of the control tissues (Figure 2a). Among the proteases involved in ACE2 mediated viral entry, *TMPRSS2* was significantly (*p* < 0.05) down‐regulated (Figure 2b), whereas *FURIN* (Figure 2c) appeared marginally up‐regulated in HNSCC but it was not statistically significant. Although OSCC represents one of most common HNSCC types, a heterogeneous group of malignancies from other areas in the head and neck are also included in HNSCC. Hence, it is important to validate the HNSCC results using datasets consisting of only OSCC. Indeed, a similar expression pattern was found for all of the genes examined in two independent OSCC microarray datasets (Figure 2d–i). Interestingly, expression profiles of the entry proteins in oral dysplastic lesions (premalignant oral lesions) were similar to that of OSCC (Figure 2d–f).

## 4 DISCUSSION

The efficiency of the coronavirus cellular entry is key for successful viral infection and pathogenesis (Perlman & Netland, 2009). The SARS‐CoV‐2 cellular entry in turn depends on the relative abundance of surface proteins ACE2, FURIN, and TMPRSS2. A recent study reported the presence of *ACE2* mRNA in normal/para‐tumor oral mucosa, especially in the TCGA dataset (Xu et al., 2020). In line with this, *ACE2* mRNA was found to be expressed in the tongue (Figure 1a), although the abundance was markedly lower as compared to other body tissues such as small intestine, colon, and kidney. The mRNA expression levels of *TMPRSS2* and *FURIN* were found to be slightly higher than that of *ACE2* in the tongue (Figure 1d,g). Of note, none of the entry proteins were immunohistochemically detected in NOM in the HPA dataset (Figure 1b,c,e,f,h,i). A lower sensitivity of immunohistochemistry as compared to the transcriptome analysis through RNA‐seq could be one of the explanations for this disagreement. In addition, the use of separate sample sets for RNA‐seq and immunohistochemistry could contribute to this discrepancy. Indeed, a similar discordance between the transcriptomic and immunohistochemistry data was reported for ACE2 and TMPRSS2 in salivary glands when using public datasets (Pascolo et al., 2020). Nevertheless, immunohistochemical expression of ACE2 (Hamming et al., 2004) and FURIN (de Cicco et al., 2004) was previously reported in oral mucosa. Overall, in the line with previous suggestion (Xu et al., 2020), these results indicate that oral epithelium can be a possible site for SARS‐CoV‐2 attachment. However, the relatively low expression levels of all entry proteins in NOM as compared to the other body tissues suggest that the risk for SARS‐CoV‐2 attachment/entry in NOM might be lower than previously suggested. The suggestion that oral cavity can be a likely site for SARS‐CoV‐2 attachment has clinical implications, as almost all procedures related to the dental examination and treatment involve close contact with saliva or salivary droplets/aerosols. Hence, it is important to follow proper hygienic measures to minimize salivary aerosol production and to use appropriate protective equipment to protect dental personnel from SARS‐CoV‐2.

Cancer patients, including OSCC are considered one of the high‐risk groups for COVID‐19 (Tian et al., 2020; Yu et al., 2020). Older age, immunocompromised status, existing comorbidities, and frequent hospital visits have been suggested be some of the factors predisposing cancer patients for SARS‐CoV‐2 infection with severe symptoms and high mortality rate (Lee et al., 2020; Yu et al., 2020). Besides these systemic/nosocomial factors, there is a growing speculation that overexpression of SARS‐CoV‐2 cellular entry proteins might contribute to amplify the risk of viral attachment in lung cancer lesions (Kong et al., 2020). The unchanged/down‐regulated expression levels of *ACE2* and *TMPRSS2* in HNSCC/OSCC indicate that their expression profiles are unlikely to confer increased risk for SARS‐CoV‐2 attachment/entry in these lesions as compared to the normal tissues. In addition, the upregulation of *FURIN* in HNSCC/OSCC in the background of unchanged *ACE2* mRNA is less likely to amplify the risk for SARS‐CoV‐2 attachment, as the pre‐primed S‐protein requires ACE2 binding for the cellular entry (Shang et al., 2020). It was interesting to note that expression profiles of entry proteins in oral dysplastic (premalignant oral lesions) lesions were similar to that of OSCC, suggesting that both oral premalignant lesions and OSCC display similar risk status for virus attachment. Taken together, the evidence is lacking to suggest that the expression status of entry proteins predisposes OSCC/oral premalignant lesions to additional risk for SARS‐CoV‐2 attachment/entry as compared to NOS.

The current study benefited from the use of protein data for NOM from the HPA, and validation of the TCGA/GTEx HNSCC data by two independent microarray datasets consisting of OSCC and normal controls from the oral cavity. The latter is especially important as HNSCC represents a heterogeneous group of malignancies not only from the oral cavity but also from the other regions in the head and neck. Additionally, control tissues in the TCGA/GTEx data mostly represent the para‐tumor tissues, and they might harbor molecular alterations (Aran et al., 2017). However, the current study also has limitations. The transcriptomic data (the FANTOM5) and proteomic data (the HPA) for NOM respectively represent only two and three specimens, and the protein data is lacking for the OSCC specimens. Future expression studies using a relatively large number of OSCC/control specimens, together with functional studies focusing on the entry mechanism of SARS‐CoV‐2 in OSCC cells are necessary to clarify the susceptibility of cancer cells to SARS‐CoV‐2 infection.

## CONFLICT OF INTEREST

The authors declare no conflicts of interest.

## AUTHOR CONTRIBUTIONS

*Conceived and designed the work*: Dipak Sapkota and Muy‐Teck Teh. *Analyzed the data*: Dipak Sapkota, Sunita Sharma, Tine M. Søland, Paulo H. Braz‐Silva, and Muy‐Teck Teh. *Wrote the manuscript*: Dipak Sapkota and Muy‐Teck Teh. *Revised the manuscript*: Dipak Sapkota, Sunita Sharma, Tine M. Søland, Paulo H. Braz‐Silva, and Muy‐Teck Teh.

## Data Availability

The data that support the findings of this study are freely available in the following public resources: the Cancer Genome Atlas (https://www.cancer.gov/about‐nci/organization/ccg/research/structural‐genomics/tcga, the Genotype‐Tissue Expression (GTEx, https://www.gtexportal.org/home/) (GTEx, 2015), the Human Protein Atlas (https://www.proteinatlas.org/) (Uhlén et al., 2015), and two microarray datasets (Chen et al., 2008; Rentoft et al., 2012).
